# Safety and Tolerability of Intramuscular and Sublingual Ketamine for Psychiatric Treatment in the Roots to Thrive Ketamine Assisted Therapy Program

**DOI:** 10.1192/j.eurpsy.2023.1264

**Published:** 2023-07-19

**Authors:** V. W. L. Tsang, B. Tao, S. Dames, Z. Walsh, P. Kryskow

**Affiliations:** 1 Psychiatry, UBC, Vancouver; 2 Health and Human Services, Vancouver Island University, Nanaimo; 3 Psychology, University of British Columbia, Kelowna, Canada

## Abstract

**Introduction:**

Ketamine has been increasingly used to treat mental health conditions yet there is a lack of safety data on intramuscular (IM) and sublingual (SL) dosing in a community setting. The Roots to Thrive Ketamine assisted Therapy (RTT-KaT) program is a 12-week program with 12 Community of Practice (CoP) group therapy sessions and three ketamine sessions.

**Objectives:**

To provide preliminary data on RTT-KAT adverse events to subsequently inform safe use of IM and SL ketamine for the treatment of psychiatric disorders.

**Methods:**

Retrospective chart review of the RTT-KaT Program on four cohorts (n=128) between September 2020 to December 2021. Eligible patients include those with post-traumatic stress disorder, depression, generalized anxiety, burnout/adjustment disorder, substance use disorder, obsessive compulsive disorder, disordered eating, and disordered sleep. Baseline characteristics and adverse events were captured including medication administration before, during, and after RTT-KaT sessions. Chi-squared test with Yates’ continuity correction was used to assess side effects in subgroups from ketamine administration.

**Results:**

RTT-KaT was well tolerated with no loss to follow up. There were 351 IM (mean dose = 102.553mg) and 96 SL (mean dose = 276.667mg) sessions of ketamine. Of the 448 sessions, the prevalence of elevated blood pressure increased by 12.31% from baseline (36.85%), with all post-treatment elevations being transient. The prevalence of elevated blood pressure post-KaT session was also similar between IM (+11.69% from 37.71% baseline) and SL (+15.12% from 32.98% baseline) administration. Regarding adverse effects, 12.05% of sessions experienced nausea , 2.52% had an episode of vomiting , 3.35% had a headache , and seven sessions experienced dizziness. The incidence of adverse events was not significantly associated with past psychedelic experiences (X2 = 0.0543, p-value = 0.8157), nor past psychiatric diagnosis (X2 = 0.0109, p-value = 0.917). . There was no significant association between administration route and incidence of nausea, which was the most common side effect(X2 = 1.112, p-value = 0.2916). Male gender was also significantly associated with lower incidence of nausea (X2 = 4.2841, p-value = 0.03847).

**Conclusions:**

The group therapy model described provides a comprehensive approach and presents a promising model for operating a KaT program outside of a clinical trial setting.These findings suggest good safety and acceptability for RTT-KaT among individuals seeking treatment for mental health issues. Majority of participants did not experience adverse reactions and the adverse events that were recorded involved transient symptoms that were resolved with rest and/or medications.

**Disclosure of Interest:**

None Declared

